# Overview of schizophrenia research and treatment in Pakistan

**DOI:** 10.1016/j.heliyon.2020.e05545

**Published:** 2020-11-23

**Authors:** Rukhsana Nawaz, Saima Gul, Rafat Amin, Tanzeel Huma, Fadwa Al Mughairbi

**Affiliations:** a Department of Clinical Psychology, College of Medicine & Health Sciences, UAE University 15551 Al Ain, United Arab Emirates; b Department of Rehabilitation Science, Faculty of Pharmacy & Allied Health Sciences, Shifa Tameer e Millat University, Islamabad, Pakistan; c Department of Pathology, Institute of Biological, Biochemical and Pharmaceutical Sciences, Dow University of Health Sciences, Ojha Campus, Karachi, Pakistan; d Quaid-i-Azam University, Islamabad, Pakistan

**Keywords:** Psychology, Psychiatry, Schizophrenia, Pakistan, Molecular genetics, Drug treatment, Mental illness

## Abstract

Mental health is the most neglected health sector in Pakistan, and the majority of citizens have limited or no access to primary and secondary psychiatric services. The incidence of schizophrenia (SCZ) has increased at an alarming rate in Pakistan, relative to that of other psychiatric disorders. While numerous studies have investigated SCZ, few have addressed the issue about the Pakistani population. In the present review, the researchers discuss current data integral to the prevalence, pathophysiology, and molecular genetics of SCZ; treatment approaches to the disease; and patient responses to drugs prescribed for SCZ in Pakistan. Most Pakistani patients exhibit poor responses to antipsychotic drugs. Based on our review, the researchers hypothesize that genetic dissimilarities between Pakistani and Western populations contribute to such poor responses. Consequently, an understanding of such genetic differences and the provision of personalized treatment may simultaneously aid in improving SCZ treatment in Pakistan.

## 1 Introduction

Schizophrenia (SCZ) is a complex neurological disorder that involves impairments in the perception or expression of reality, accompanied by significant social or occupational dysfunction [1, 2]. The symptoms of SCZ can be classified into four broad categories: positive symptoms, negative symptoms, cognitive impairment, and other mood disturbances [3, 4]. Such symptoms include hallucinations, delusions, disordered thinking, movement disorders, flat affect, social withdrawal, and cognitive deficits. Patients with SCZ may also experience fearfulness, withdrawal, extreme agitation, sleep disturbances, and behavioral changes [5]. Besides, speech content is often bizarre in patients with SCZ, who may also sit for hours without moving or talking, or exhibit no signs of impairment until they begin speaking about their thoughts. Although it is a common misconception, SCZ is not characterized by dissociative identity disorder, where patients exhibit episodic rather than continued dysfunction [6].

Formal operational criteria used in the diagnosis of SCZ are outlined in the International Classification of Diseases-10 (ICD-10)—recommended by the World Health Organization in 1992—and the 5^th^ edition of the Diagnostic and Statistical Manual of Mental Disorders (DSM-5). According to ICD-10 criteria (the official system used for clinical diagnosis in European countries), symptoms should be present for at least 1 month. In contrast, DSM criteria are frequently used in the United States, specifying that symptoms must be present for at least 6 months [7]. Regardless of this discrepancy, a high level of agreement exists between the two classification systems.

## 2 Prevalence of Schizophrenia

SCZ is among the top ten causes of disability worldwide, affecting approximately 1% of the global population (approximately 24 million people). Nevertheless, more than 50% of these individuals do not receive appropriate care [8]. The socio-economic burden of SCZ is devastating for patients, their family members and friends, the healthcare system, and, ultimately, the country. Onset during early adulthood, the need for a lifelong treatment course, the lack of social acceptability, and the debilitating nature of its symptoms collectively render SCZ one of the most disabling and financially exhausting disorders [8]. SCZ onset usually occurs between the ages of 15 and 40 years and affects men and women with equal frequency. But, in some cases, the onset of the disorder occurs later in women than in men [9].

Variation in the prevalence of SCZ across geographic regions, populations, and ethnic groups has been suggested but not confirmed [10]. Approximately 90% of patients with SCZ who apparently and undoubtedly reside in low- and middle-income/Third World countries such as Pakistan (www.who.int/mental_health/management/schizophrenia/en/). While the exact prevalence of SCZ in Pakistan remains unknown, a report by Akhtar [11] indicated that approximately 1.5% of the Pakistani population is affected by SCZ. Taking into consideration the global prevalence of SCZ and the findings presented in the draft of the “Assessment of Health Status & Trends in Pakistan,” the estimated prevalence of SCZ in Pakistan may be as high as 1–2% of the total population [12]. However, estimates of SCZ prevalence vary greatly about rural and urban populations in various provinces. In Punjab, the prevalence is estimated to be 2.5% among people in urban areas and 2% among people in rural areas. In Sindh, it is estimated to be 2% among the urban population and 1.5% among the rural population. In Khyber Pakhtunkhwa, the prevalence of SCZ is estimated to be 2% among the urban population and 2.5% among the rural population. Baluchistan has the lowest estimates, with an estimated prevalence of 1% for both rural and urban populations [13].

In addition to genetic makeup, various factors such as drug-addiction, underlie the predisposition toward SCZ. A large number of people in Pakistan, mostly men, are addicted to tobacco, cannabis, betel nut, charas, and other illicit drugs [14]. Repeated administration of these stimulants at a younger age (<15 years) increases the likelihood of developing SCZ [15]. This finding is consistent with those of another study, which suggested that the proportion of Pakistani people who use cannabis and exhibit schizophrenic symptoms is significantly higher among men than women. This may be explained by the fact that cannabis increases levels of dopamine in the brain, which can, in turn, lead to schizophrenic symptoms [16].

Pakistani men are more susceptible to SCZ than their female counterparts. The high susceptibility among men may be due to their higher exposure to pollutants and daily stress associated with work, which is known to trigger psychotic symptoms in patients with a genetic disposition toward psychosis [16]. Unlike men, women in Pakistan usually begin to exhibit symptoms of SCZ after marriage or during/after the birth of a baby. Previous studies have demonstrated that levels of the amino acid tryptophan are lower during the postpartum period, resulting in lower overall levels of serotonin and leading to depression [17]. A recent study further reported that the hormone estrogen is involved in memory and cognition and that imbalances in estrogen levels may impact cognitive function in the mother or influence cognitive development in the fetus [18].

## 3 Pathophysiology of Schizophrenia

SCZ is far more multifaceted than originally assumed, involving a combination of genetic factors and structural abnormalities. According to the neurodevelopmental model of SCZ, silent lesions in brain regions conflated with the development of integration among the frontal, parietal, and temporal cortices arise mainly during the very early stages of brain development, primarily in the prenatal or early postnatal period of life [19]. These lesions do not strongly interfere with basic brain functions in the early years of life, though symptoms may manifest during adolescence and young adulthood. Such a transition occurs when the individual is stressed by increasing demands connected with the functional integration of the aforementioned brain regions during the formative years. Previous studies have reported that patients with SCZ may exhibit third ventricle enlargement, as well as abnormalities in the medial temporal lobe, amygdala, hippocampus, and neo-cortical temporal lobe [20]. Additional studies have revealed that patients with SCZ exhibit atrophy in the hippocampus due to hyper-metabolism, which is an early sign of this disorder [21].

In relation to Pathophysiology of SCZ, number of studies revealed that the possible role of altered brain long-range functional interactions underlying the link between aberrant self-experience and self-other relationship [22]. Ebisch et al. reported that Self-experience anomalies are elementary features of schizophrenic pathology. They provide a link between aberrant self-experience and social cognition in first-episode schizophrenia (FES). Further, their study concludes that an imbalance in the processing between internally and externally guided information and its abnormal integration with self-referential processing as mediated by posterior cingulate cortex (PCC). This imbalance closely relates to basic symptoms in FES and thus, anomalous self-experience [23]. These changes in brain morphology are thought to be the main cause of SCZ.

## 4 Role of Neurotransmitters

Various researchers have postulated a link between altered brain function and SCZ. The most influential and plausible theory signifies that disturbed neuronal circuitry in the brain causes irregularities in brain activity and sensory processing [20]. Emerging evidence suggests that neurotransmitters play an important role in the etiology of SCZ and that neural network connectivity is altered by the disruption of synaptogenesis and neuroplasticity [24].

*Glutamate* is an excitatory neurotransmitter that mediates synaptic transmission in the central nervous system (CNS). The association between glutamate and SCZ was first hypothesized in the 1980s [25]. Studies have tended to demonstrate that hypo-functionality of glycine and glutamate receptors (NMDAR) in the frontal lobe and hippocampus is associated with the positive symptoms of SCZ [26, 27]. Given the importance of neurocognitive dysfunction in the conceptualization of the disorder, post mortem studies have suggested that SCZ is associated with decreased NMDAR function [24, 25]. Indeed, glutamate-blocking agents have been observed to improve cognition and decrease positive and negative symptoms in patients with SCZ [24, 25]. Besides, glutamate regulates the release of dopamine from the prefrontal cortex to the ventral tegmental area via the mesolimbic pathway. This activity of glutamate in the frontal lobe is critical for the lower basal release of dopamine [27].

*Dopamine* is an excitatory neurotransmitter in the CNS that aids in the regulation of emotions and body movement. Abnormal dopamine levels are associated with certain neurological disorders, such as Parkinson's disease. The dopamine hypothesis proposes that the symptoms of SCZ are due to the hyperactivity of dopaminergic receptors. A recent meta-analysis has revealed that the brains of patients with SCZ exhibit increased D2 receptor density and dopamine content [28]. Dopaminergic activity in the prefrontal cortex, thalamus, and striatum are also affected in patients with SCZ [29]. Following this finding, Jean Claude et al. [29] revealed that altered levels of D2 receptor binding in the thalamus and striatum may be implicated in SCZ.

The symptoms of SCZ can be reduced by medications that block D2 receptors. Recent evidence has indicated that untreated patients with SCZ exhibit decreased D2 binding potential in the thalamus as well as increases in the number of D2 receptors in the striatum [29]. Another study by Moghaddam and Adam [30] revealed that a low dose of ketamine increased dopamine release in the prefrontal cortex. Animal studies have also demonstrated that temporary overexpression of D2 receptors in transgenic mice leads to abnormal function of the prefrontal cortex. Furthermore, knockdown of dysbindin siRNA—which modulates D2 receptor internalization—results in decreased glutamate release in primary neurons [31]. Unequivocally, the previous studies have suggested that decreased exocytosis of glutamate-containing synaptic vesicles can lead to decreased dysbindin levels, which alter neuronal transmission and may be associated with the clinical symptoms of SCZ.

*Serotonin* (also known as 5-hydroxytryptamine or 5-HT) appears to be involved in the pathophysiology of psychosis. Serotonin has been conflated with a variety of behaviors and somatic functions, such as cognition, memory, perception, sensory gating, mood, aggression, pain sensitivity, endocrine function, and sleep [32]. Many of these functions are linked to the etiology of positive and negative symptoms and with cognitive impairments in patients with SCZ [33]. Functional alterations in the serotonergic system at the pre- and postsynaptic levels can affect multiple neurotransmitter systems (e.g., glutamate, GABA, norepinephrine, acetylcholine, and dopamine). Enhanced neurotransmission of dopamine and serotonin in subcortical areas has been coupled with the positive symptoms of SCZ. In contrast, decreased activity of both dopamine and serotonin in the prefrontal cortex has been associated with the negative symptoms of SCZ [34, 35, 36].

These findings have led to the use of a combination of serotonin antagonists with either partial D2 antagonists or glutamate receptor (mGlu2/3 receptor) agonists. These combinations have proven very effective in the treatment of the positive, negative, and cognitive symptoms of SCZ [37, 38, 39, 40, 41].

Gamma-aminobutyric acid *(GABA)* is a major inhibitory neurotransmitter in the brain. Decreased GABA production has been observed in various conditions, such as insomnia, epilepsy, and anxiety disorders. Research has indicated that patients with SCZ exhibit decreased expression of GABA transcripts that encode GABA neurons, and that this decrease in the level of GABA results in working memory dysfunction and the disruption of central sensory gating mechanisms. Thereupon, individuals with SCZ are unable to filter out extraneous information, which results in an over-inclusive thought pattern and difficulty concentrating [42, 43]. Dysfunction of proper GABAergic inhibition in the cerebral cortex underlies the pathophysiological process of SCZ [42, 44, 45].

Further investigation of the neurophysiological abnormalities that occur before and during SCZ is critical, not only for elucidating the etiology of the disorder but for the development of novel pharmaco-therapeutic targets with improved tolerance and efficacy.

## 5 Genetics of Schizophrenia

SCZ typically manifests during late adolescence or early adulthood. Early-onset of the disorder suggests that genetic vulnerability is the major determinant, whereas environmental factors help to mediate cellular events that amplify the genetic predisposition and increase the risk of developing SCZ. Encounters with social and economic stressors or the abuse of addictive drugs during puberty may increase susceptibility to psychiatric disorders. Collectively, these factors can cause aberrations in brain development and synaptic connections that may result in emergent schizophrenia [46, 47, 48].

It has been well documented that mental illnesses aggregate in families. Based on evidence from multi-disciplinary research studies, the heritable component of SCZ may be as high as 80% [49, 50]. Numerous family, twin, and adoption studies have demonstrated that individuals whose first-degree relatives have SCZ (parent, sibling, or offspring) exhibit a 5–10% increase in the risk of SCZ, relative to the 1% observed in the general population [51, 52]. This risk increases to 13% for a child who has one parent with SCZ, and to 35–40% for a child who has two parents with SCZ [53].

Due to its high heritability and strong familial associations, genetic approaches are critical in the investigation of SCZ etiology. Initial studies of the genetic mechanism underlying SCZ have concluded that SCZ is genetically transmitted from parents to their offspring [54, 55, 56, 57, 58]. Belying early expectations, increasing evidence suggests that the causes of SCZ are not associated with a single mutation of a single gene. Rather, multiple DNA variants, not all of which have been identified, are involved in SCZ. Thus, the additional risk may be the result of interactions between individual DNA variants and different environmental factors that underlie the individual variations of SCZ.

Both family and adoption studies have suggested that several genetic loci are involved in the genesis of SCZ [59]. Linkage analyses of schizophrenic genes that have been performed on chromosomes 2q [60], 3p [61], 4p [62], 5q [63]^,^ and 22q [64,65] around the world whereas the first study from Pakistan performed haplotype and linkage disequilibrium analysis association of DISC locus (1q24.1) with a risk of schizophrenia and they reported the effect of human genome region 1q24.1 in transmission and liaison of schizophrenia in Pakistani population [66, 67]. Scientist Fatima and her colleagues identified that the 325 Kb region on 1q24.1 can be considered a prognostic marker for schizophrenia development in the Pakistani population [66].

Various genres such as *AKT1, BDNF, CAPON, CCKAR, CHRNA7, CNR1, COMT, DNTBP1, GAD1, GRM3, IL10, MLC1, NOTCH4, NRG1, NR4A2/NURR1, PRODH, RELN, RGS4, RTN4/NOGO*, and *TNF*-α have been associated with SCZ in different populations worldwide [68, 69, 70]. Previously, there were four discovered susceptibility genes *TSNARE1*, *PBRM1*, *STAB1*, and *OLIG2,* also with four novel susceptibility loci *PSEN1*, *TLR5*, *MGAT5B*, and *SSPO* discovered in Han Chinese SCZ patients [71]. Mutations in these genes completely disrupt the ability of the neural circuitry to respond to the abnormal secretion of neurotransmitters, leading to changes in behaviour [72].

Biodiversity and genetic variations constitute the best example of single nucleotide polymorphisms (SNPs), which normally function to retain genetic variety in a population living in a varied environment. Therefore, to confirm gene diversity and its association with SCZ, researchers have investigated SNPs intrinsic in SCZ, identifying many susceptible genes in various populations [73]. An exclusive association of different SNPs in the susceptible genes of SCZ has been identified in the Pakistani population. Some of these SNPs do not exhibit an association with SCZ in the Pakistani population compared with other Asian and Western populations [74, 75]. The neuregulin 1 (*NRG1*) gene has been associated with SCZ in several populations [76, 77, 78, 79, 80]. However, published studies have demonstrated an association between *NRG1* and *TNF*-α gene mutations and SCZ in the Pakistani population [81]. Earlier, Nawaz et al [14] and Naqvi et al [82] demonstrated that the *NRG1* genetic variant SNP rs35753505 and polymorphism8nrg433E1006 plays an important role in conferring susceptibility to SCZ in the Pakistani population and may represent a potential antipsychotic drug target in such patients [15].Moreover, Amir et al provided strong evidence that supports the role of *CACNA1C*, *GRM3*, and *DRD2* genes as schizophrenia susceptibility genes in the Pakistani population [74]. Nevertheless, one study has shown that some genetic variants rs1801028 and rs6277 of DRD2 are not associated with schizophrenia in the Pakistani population [83]. Further investigations in the field of neurogenetics and pharmacogenetics are essential to the improvement of our understanding of the molecular etiology of SCZ and the design of effective drugs based on variations in the genetic makeup of the Pakistani population.

## 6 Schizophrenia Treatment

The treatment of patients with SCZ is challenging because the pathophysiology of the disorder is not well understood. In high-income countries, SCZ is best treated in specialty clinics. The principal intentions in the treatment of SCZ are to decrease the incidence and severity of psychotic exacerbation, ameliorate positive and negative symptoms, and increase patients’ functional aptitude and quality of life. The priority of neuropsychiatric practitioners is therefore to identify patients in the early stages of psychosis and provide the recommended treatment. Clinicians encounter and address the diagnosis in a variety of contexts, including initial presentation, provision of support to family members, evaluation of concurrent medical illness, management of medications and their side effects, and primary treatment when specialty options are not available. Clinicians in high-income countries also offer convenient and non-stigmatizing access to health care for patients affected with SCZ-related disorders [84].

However, few psychiatric and specialty clinics for the diagnosis and treatment of SCZ exist in Pakistan. Similarly, few studies have focused on understanding and treating Pakistani patients with SCZ [13]. In this region, mental health care depends heavily on the use of medications (antipsychotic drugs) and medical and physical therapies, including electro-convulsive therapy (ECT). In general, SCZ is treated using antipsychotic drugs, to correct neurotransmitter imbalances [85]. Such drugs are known to block the behavioral effects of phencyclidine (PCP) and a variety of neurotransmitter receptors, such as the NMDA-glutamate ion channel, thus decreasing positive and negative symptoms [86]. Dopamine super sensitivity psychosis in schizophrenia can be developed by long-term antipsychotic treatment and DPS may play a vital role in the process leading to treatment recalcitrance in schizophrenia [87, 88]. Moreover, a multi receptor atypical antipsychotic clozapine is also approved for the treatment of resistant schizophrenia but despite its proven efficacy to SCZ treatment, some populations, i.e., elderly and adolescents, may be particularly vulnerable to clozapine adverse effects [89].

Antipsychotics primarily deal with psychosis and managing the symptoms of the disorder, enabling patients to function in daily life. However, survey-based studies [90, 91] revealed that the majority of Pakistani patients prefer to pursue medical and religious healing therapies simultaneously, and some choose to forgo psychiatric interventions entirely and seek healing only from religious sources [90, 91]. Therefore, further research is required to examine the knowledge and practices of general practitioners in different districts across Pakistan. Psychiatrists and researchers should aim to highlight the magnitude of the issue, to address the gaps in knowledge and practice in this context, and to make improvement plans if required. Moreover, there is a need to examine the effect of psychosocial education regarding SCZ, as such education may lead to increased awareness of the various treatment options.

The Eastern cultural lifestyle centres on religious beliefs and activities. Seeking help from faith healers and visiting religious destinations for a variety of health issues are common practices. Piraniin [92] conducted a qualitative ethnographic study of individuals who had visited a Muslim shrine in search of healing for “mental suffering”. According to this report, thousands of people visit shrines in search of help and healing in times of need, including those who desire better health outcomes [93, 94]. A review of the literature indicates that those who visit shrines have often been diagnosed with psychiatric disorders [95]. For individuals and families dealing with mental health problems, religious healing is often the first line of defense [96, 97, 98].

## 7 Schizophrenia Research in Pakistan

Antipsychotic drugs are the treatment of choice for SCZ throughout the world. Patients with SCZ receive greater benefit from the combined use of antipsychotic drugs and psychosocial intervention than from pharmacotherapy alone. This combined treatment costs less than the standard treatment and is suitable for psychiatric rehabilitation [99].

Formative research on first-episode psychosis began in the 1980s [100, 101]. The first clinical service for psychosis was introduced in Melbourne [102] and quickly spread to many locations in Europe, the United Kingdom, Asia, and North America [103]. There are now more than 100 intervention programs operating worldwide, with a particular focus on children and their family members. The Royal Australian and New Zealand College of Psychiatrists has published international clinical practice guidelines for the treatment of SCZ, particularly concerning early psychosis [104]. This organization has 3,000 members from different countries who are working to increase awareness of and improve clinical care and facilities for early psychosis. Recently, the National Institute of Mental Health (NIMH) in the United States announced that it will increase funding for the study of first-episode psychosis and the development of more effective services for affected patients [105].

In Pakistan, physicians and psychiatrists based their practices on cross-sectional studies. Most of these studies have examined prescribing preferences for antipsychotic drugs. Health care professionals prefer antipsychotic drugs based on years of clinical experience, cost, and availability [90]. However, these studies have provided no strong evidence regarding the efficacy of particular dosages, concentrations, and/or effects on neurotransmitter levels (increase or decrease). Clinicians normally do not assess neurotransmitter levels before or after prescribing antipsychotic drugs, and most professionals do not know the molecular mechanism underlying the actions of these drugs.

In low- and middle-income countries such as Pakistan, the nature of clinical experience is very different from that in high-income countries [91]. To better understand the socio-cultural context of SCZ, further research must be conducted in Pakistan. Professionals and psychiatrists should be trained to conduct clinical trials of any newly marketed antipsychotic drug in the local population. These clinical trials will help psychiatrists and professionals in evaluating which drugs are most efficient in managing the SCZ, reducing symptoms with fewer side effects, and placing a lower financial burden on affected patients and their families. Apart from pharmacological intervention, psychotherapy usefulness as a stand alone or in combination with regular pharmacological treatment had been tested with different results. Number of meta-analysis suggested that CBT in combination with cognitive remediation lead to an enhancement of therapeutic effects [106]. use number for this reference. On the other hand, other studies suggested that the effect of CBT on the core symptoms of SCZ is minimal [107].

In Pakistan, Naeem et al. (2015) tested the effectiveness of CBT in targeting the symptoms of SCZ. In their study, brief culturally adapted CBT for psychosis (CaCBTp) plus treatment as usual (TAU) was administered (n = 59) & compared the results with that of TAU alone (n = 57). The CaCBTp sessions were provided over 4 months period, and SCZ symptoms were measured using PANSS (Positive & Negative Syndrome Scale of Schizophrenia) among other psychological measures. Their results suggested that combining CaCBTp with regular pharmacological treatment can be effective in low & middle income countries such as Pakistan. However, there is no evidence provided for long term effect of the intervention when compared to the TAU alone. It is clear that more randomized control trials examining the long-term efficacy of CBT for psychosis are needed [108].

## 8 Patient Response in Pakistan

Pakistani patients with SCZ do not respond uniformly to antipsychotic medication concerning the reduction of symptoms and side effects. A total of 30–40% of patients with SCZ are regarded as poor responders to antipsychotic treatment. Given that a familial pattern of treatment response has been observed, genetic factors may be inherent in such variations in treatment response [109]. Furthermore, ethnic heterogeneity may determine atypical responses to antipsychotic drugs among the Pakistani population. Pharmacogenetic researchers focus on variations in the human genome and ways in which genetic factors may influence individual responses to drugs. Some genetic variants alter the risk of dependence on one drug, whereas others affect responses to various drugs [109]. Additionally, genetic studies and geno-phenotypic relationships indicate that the relative contributions of genetic and non-genetic components (e.g., environmental) can impact a particular phenotype. Brain physiology associated with responses to antipsychotic drugs also differs from person to person. While some patients may experience side effects such as skin rashes, drowsiness, headache, dizziness, rapid heartbeat, menstrual problems, weight gain, increased risk of diabetes/high cholesterol, etc.) when taking antipsychotic medications, others experience no side effects or complications.

In Pakistan, non-compliance is common [110, 111]. The most common reasons for non-compliance include a lack of awareness regarding the benefits of treatment, an inability to afford the drugs, physical side effects, a lack of awareness among doctors, and the stigma surrounding mental health disorders and treatment. In Pakistan, drug non-compliance has been a major hindrance to the effective management of SCZ. Nearly 74% of patients experience a relapse of the illness and require frequent readmission due to non-compliance [110, 111]. Various studies provided evidence that long-term antipsychotic treatment can minimize relapse and provide clinical benefit to SCZ patients [112]. These studies also suggest that Long-acting injectable formulations of second-generation antipsychotics (SGAs-LAIs) provide constant medication delivery and the potential for improved adherence [113].

Therefore, Psychiatry Departments should develop programs for supervised treatment in outpatient clinics. Moreover, future studies should aim to compare the effectiveness of supervised treatment and standard outpatient care and to identify and address factors associated with non-compliance to facilitate the development of a comprehensive treatment plan.

## 9 Conclusion

Few studies conducted in Pakistan have investigated target-gene-environment interactions at the molecular level in patients with SCZ. Thus far, no study has used drug trials to identify better treatment options, and psychiatrists and other healthcare professionals usually follow simple health care strategies to reduce symptoms.

In this review, the researchers aimed to highlight these issues and demonstrate how clinical and genetic investigations may overcome current challenges. Further research may have an impact on service design and positive treatment outcomes. In Pakistan, patients do not have access to psychiatric services at the clinical level, and physicians do not have data to predict patients’ responses to a specific drug. These factors impact the assessment of functional outcomes in youth at risk for psychosis as well as individual patient responses, drug efficacy, side effects, and affordability.

According to the existing literature, environmental and economic factors have little or no major impact on SCZ, while genetic diversity and population-level mutations likely form the biological basis of SCZ [109]. Therefore, it is important to investigate these genes and their diversity, which has previously been shown to exhibit a strong correlation and association with SCZ in Western populations.

Practicing neuropsychiatric physicians in Pakistan follows the recommendations of Western clinicians regarding the dosage of antipsychotic drugs. Nevertheless, most of their patients exhibit poor responses, and symptoms re-materialize in some patients after medications have been discontinued. Few studies have investigated the poor response to antipsychotic medicines in the Pakistani population in comparison with Western populations. Additional research is needed to investigate the molecular genesis of this poor response, and to build a foundation for providing personalized antipsychotic treatment that maximizes the probability of good responses and minimizes side effects.

## Declarations

### Author contribution statement

All authors listed have significantly contributed to the development and the writing of this article.

### Funding statement

This research did not receive any specific grant from funding agencies in the public, commercial, or not-for-profit sectors.

### Data availability statement

No data was used for the research described in the article.

### Declaration of interests statement

The authors declare no conflict of interest.

### Additional information

No additional information is available for this paper.
